# Use of Electrosprayed Agave Fructans as Nanoencapsulating Hydrocolloids for Bioactives

**DOI:** 10.3390/nano8110868

**Published:** 2018-10-23

**Authors:** Jorge A. Ramos-Hernández, Juan A. Ragazzo-Sánchez, Montserrat Calderón-Santoyo, Rosa I. Ortiz-Basurto, Cristina Prieto, Jose M. Lagaron

**Affiliations:** 1 Laboratorio Integral de Investigación en Alimentos, Tecnológico Nacional de México/Instituto Tecnológico de Tepic, Av. Tecnológico 2595, C.P. Tepic 63175, Nayarit, Mexico; jorgeramos17@outlook.com (J.A.R.-H.); montserratcalder@gmail.com (M.C.-S.); riobasurt@ittepic.edu.mx (R.I.O.-B.); 2 Novel Materials and Nanotechnology Group, IATA-CSIC, Calle Catedrático Agustín Escardino Benlloch 7, 46980 Paterna, Spain; cprieto@iata.csic.es (C.P.); lagaron@iata.csic.es (J.M.L.)

**Keywords:** HDPAF, β-carotene, electrospraying, encapsulation, photoprotection

## Abstract

High degree of polymerization Agave fructans (HDPAF) are presented as a novel encapsulating material. Electrospraying coating (EC) was selected as the encapsulation technique and β-carotene as the model bioactive compound. For direct electrospraying, two encapsulation methodologies (solution and emulsion) were proposed to find the formulation which provided a suitable particle morphology and an adequate concentration of β-carotene encapsulated in the particles to provide a protective effect of β-carotene by the nanocapsules. Scanning electron microscopy (SEM) images showed spherical particles with sizes ranging from 440 nm to 880 nm depending on the concentration of HDPAF and processing parameters. FTIR analysis confirmed the interaction and encapsulation of β-carotene with HDPAF. The thermal stability of β-carotene encapsulated in HDPAF was evidenced by thermogravimetric analysis (TGA). The study showed that β-carotene encapsulated in HDPAF by the EC method remained stable for up to 50 h of exposure to ultraviolet (UV) light. Therefore, HDPAF is a viable option to formulate nanocapsules as a new encapsulating material. In addition, EC allowed for increases in the ratio of β-carotene:polymer, as well as its photostability.

## 1. Introduction

Fructans from *Agave tequilana* consist of a complex mixture of fructooligosaccharides (fructose polymer obtained by enzymatic hydrolysis of high polymerization degree Agave fructans (HDPAF) by fructan exohydrolase (FEH) and 1-fructosyl transferase (1-FFT enzymes) containing principally β-(2→1) fructosyl–fructose linkages, but also β-(2→6) and branch moieties) [1,2]. The physico-chemical and functional properties of fructans are linked to the degree of polymerization (DP) as well as the presence of branches. The short-chain fraction, oligofructose, is much more soluble and sweeter than native and long-chain fructans, and can contribute to improve mouthfeel because its properties are closely related to those of other sugars. The high DP (>40 fructose units, *M*w = 3259.95 ± 181.75 g/mol) fraction can be used as a fat substitute in low-fat or reduced-fat products (i.e., baking, ice-cream, beverages and yoghurt) since it is less soluble, more viscous and more thermostable than native fructans, which allows for modification of the rheological and sensorial properties of dairy products. In this case, fructans act as a filler or as a breaker of structure in the same way as fat globules do [1,3,4].

To our knowledge, little work has been done on the exploration of the technological applications of fructans. In this way, Furlán et al. [5] evaluated high, medium and low polymerization degree Agave fructans from *Agave tequilana* Weber as lyoprotectant agents on bovine plasma proteins during spray drying and storage. They concluded that the Agave fructans were able to cryoprotect food proteins. Thus, Agave fructans are a valuable alternative as a functional ingredient for food formulation. Ortiz-Basurto et al. [6] studied the characteristics and applications of medium and high polymerization degree Agave fructans from *Agave tequilana* Weber as microencapsulating materials of pitanga or Surinam cherry (*Eugenia uniflora* L.) juice by spray drying. The powders from both fractions were stable and able to protect the bioactive compound during and after the spray-drying process. These good results, together with its characteristics as a biopolymer (classified as biodegradable and Generally Recognized as Safe GRAS [7]), make fructans a really interesting encapsulating material for food, pharma and cosmetic applications.

Up until now, several techniques have been used to encapsulate bioactive components for the food industry, such as extrusion methods [8], fluidized bed coating [9], spray cooling [10] or spray drying [11]. Nowadays, spray drying is the most common and cheapest technology in the food industry to produce microencapsulated additives for food applications [12]. The electrohydrodynamic processing, including both electrospinning and electrospraying techniques, has recently arisen as an alternative technology that can also be used for encapsulation [13,14]. The basic setup for electrospraying consists of four main components: (1) a high-voltage source (1–30 kV), usually operated in direct current mode, though alternating current mode is also possible, (2) a blunt-ended stainless steel needle or capillary, (3) a syringe pump, and (4) a grounded collector in the form of a flat plate. The electrospraying process involves the application of a strong electrostatic field between two electrodes and imposed on a polymer solution. When increasing the electrostatic field up to a critical value, charges on the surface of a pendant drop destabilize the shape of the solution from partially spherical to conical, i.e., the so-called Taylor’s cone effect. As the charged jet accelerates toward regions of lower potential, the solvent is evaporated [15]. Besides being a very simple technique, the solvent is evaporated at room temperature; thus, it constitutes an ideal method for protecting sensitive encapsulated ingredients.

The aim of this work was to study the ability of fructans to form capsules by electrospraying and to asses, as an example, the viability of this polysaccharide as encapsulating material. For that purpose, β-carotene was selected as a model substance. The produced particles were characterized in terms of morphology and photoprotective effect.

## 2. Materials and Methods

### 2.1. Materials

High polymerization degree Agave fructans (HDPAF) were purchased from Campos Azules Co., (Ciudad de Mexico, Mexico). TEGO SML (sorbitan fatty acid ester) was purchased from Evonik Inc., (Essen, Germany). HPLC grade methanol, absolute ethanol and β-carotene were purchased from Sigma-Aldrich (Steinheim, Germany). Deionized water was used throughout the study.

### 2.2. Preparation of Formulation

In order to demonstrate the ability of HDPAF to form nanocapsules, different solutions and emulsions were prepared. Solutions contained different concentrations of HDPAF (5%, 10%, 20%, 30%, 40% and 50% *w*/*w*), TEGO SML (1%) as a surfactant and a hydroalcoholic solution (water–ethanol, 9:1) as a solvent. They were prepared under magnetic stirring at 350 RPM for 5 min (Agimatic-S model 7000242). Oil in water emulsions (O/W) were formulated at a ratio of 10:90. The continuous phase consisted of different HDPAF concentrations (4%, 9%, 19%, 29%, 39% and 49%) dissolved in the hydroalcoholic solution (water–ethanol, 9:1). The dispersed phase consisted of extra virgin olive oil and was used without further processing. TEGO SML (5% of total emulsion volume) was used as a surfactant to aid the emulsion stability and decrease surface tension. The two phases were first mixed in a high-shear mixer at 16,800 RPM for 2 min (Ultra Turrax T25, IKA, Staufen, Germany) in order to prepare the pre-emulsion. The emulsion process was carried out with an ultrasonic homogenizer model Sonopuls 2200 (Bandelin Electronic Gmbh & Co., Berlin, Germany) at 20 kHz for 1 min, according to Paximada et al. [16]. The temperature was maintained at 25 ± 1 °C using an ice bath.

The ability to electrospray the solutions and emulsions was evaluated, parameters (flow-rate (30–50 μL/h), voltage (10–20 KV) and the tip-to-collector distance (10–25 cm)) were varied one at a time until the Taylor’s cone was visible, and then particle morphology and size were analyzed to select the most adequate solution and emulsion for the photoprotection study.

For solutions containing β-carotene, β-carotene (0.1%) was incorporated in ethanol and then mixed with the solution containing water, TEGO SML and HDPAF. The mixtures were homogenized under continuous stirring at 350 RPM for 30 min. For the emulsions, β-carotene (1%) was previously incorporated in dichloromethane (1 mL) and was gradually added to the olive oil. When the oily phase was saturated with β-carotene, dichloromethane was separated for 24 h by natural evaporation in the extraction chamber, and then the mix was incorporated into the ethanol. The oily phase was added to the solution containing water, TEGO SML and HDPAF, following the same emulsion preparation procedure previously stated.

### 2.3. Characterization of Different Solutions and Emulsions

The apparent viscosity (η) was determined using a rotational viscosimeter Visco Basic Plus L from Fungilab S.A. (San Feliu de Llobregat, Spain) with a Low Viscosity Adapter (LCP). The LCP spindle was placed in the runner bar of the viscometer and 50 mL of sample was placed in a Falcon tube and put in contact with the spindle to obtain the η value. The surface tension was measured using the Wilhemy plate method in an EasyDyne K20 tensiometer (Krüss GmbH, Hamburg, Germany). Twenty-five milliliters of sample was placed in a vessel, then the Wilhemy plate is burned and suspended from the pendulum; the vessel is then placed on the platform to be analyzed. The conductivity was measured using a conductivity meter XS Con6 (Labbox, Barcelona, Spain). The probe was submerged in 10 mL of sample in a Falcon tube until the sensors were covered and stabilized. All measurements were made at 25 °C in triplicate.

### 2.4. Preparation of Capsules by Electrospraying

The electrospraying apparatus, equipped with a variable high-voltage 0–30 kV power supply, was a Fluidnatek^®^ LE-10 from BioInicia S.L. (Valencia, Spain). Solutions and emulsions with and without β-carotene were introduced in a 12 mL plastic syringe and were electrospun under a steady flow rate using a stainless-steel needle of 700 µm diameter. The needle was connected through a Polytetrafluoroethylene (PTFE) tube to the syringe. The syringe was lying on a digitally controlled syringe pump while the needle was horizontal towards the collector. The electrospraying conditions of the solutions and emulsions for obtaining the capsules were optimized and fixed at 0.1 mL/h of flow-rate, 17 kV of voltage and a tip-to-collector distance of 22 cm. The samples were stored in darkness until analysis.

Additionally, a different encapsulation strategy, named electrospraying coating (EC), patented by Lagaron et al. [17] and reported by Librán et al. [18], was used. The coating was a three-step process carried out at room temperature. In the first step, an initial layer of fructans were electrosprayed over the collector. Secondly, 2% of β-carotene with respect to the solution electrosprayed was spread out over the initial electrosprayed material layer. Finally, a top coating layer of fructans was electrosprayed directly over the material to achieve full encapsulation. The capsules were then collected and mechanically mixed and homogenized. The basic setup of a Fluidnatek™ LE10 (Bioinicia S.L., Valencia, Spain) was used to conduct the electrospraying process. The collected nanocapsules were stored in a desiccator at 0% relative humidity (RH) and protected from light for subsequent analysis.

### 2.5. Scanning Electron Microscopy (SEM)

The morphology and size of the encapsulation structures were examined using SEM on a Hitachi microscope (Hitachi S-4100, Tokyo, Japan) after having been sputtered with a gold–palladium mixture under vacuum for 3 min (SC7640, Polaron, Kent, UK). All SEM experiments were carried out with 1–2 mg of sample at 10 kV, obtaining three micrographs per sample. Capsule diameters were measured by means of the Adobe Photoshop CS3 software from the SEM micrographs in their original magnification.

### 2.6. Fourier Transform Infrared Spectroscopy

Attenuated Total Reflectance Fourier Transform Infrared spectroscopy (ATR-FTIR) (Thermo Scientific Nicolet, iS5 iD5, Waltham, USA) was used to evaluate β-carotene, empty HDPAF nanocapsules and nanocapsuled β-carotene. The samples were placed onto the ATR crystal and all the spectra were recorded from 600 cm^−1^ to 4000 cm^−1^ with a resolution of 8 cm^−1^.

### 2.7. Thermogravimetric Analysis (TGA)

Thermogravimetric analyses of free β-carotene and HDPAF nanocapsules without and with β-carotene were done in triplicate using TGA 550 equipment (TA Instruments, New Castle, USA) and TRIOS 4.3.0.38388 was the analysis software used. The analyses were conducted under the following conditions: 3–6 mg of sample, heating from 25 °C to 500 °C, at a heating rate of 5 °C/min under nitrogen flow.

### 2.8. Ultraviolet (UV) Photostability

With the aim of accelerating the oxidation of β-carotene and simulating the radiation of natural sunlight, an Osram Ultra-Vitalux (300 W) lamp (OSRAM, Múnich, Germany) was used. This blend of radiation is generated by a quartz discharge tube and a tungsten filament [19]. Nanocapsules with β-carotene and free β-carotene were exposed to the UV radiation (13.6 W) at 37 °C. After irradiation at different times (0 h, 6 h, 12 h, 24 h and 48 h), extraction of β-carotene from 2.5 mg of nanocapsules was carried out. The polymeric capsule wall was opened with water (1 mL) under magnetic stirring (200 RPM, 1 min). β-carotene was extracted from the mixture by adding 0.75 mL of chloroform and separated by centrifugation (10,000 RPM, 1 min). An aliquot of the organic phase was taken and the absorbance was measured at 466 nm in a spectrophotometer (Spectrophotometer UV/VIS4000, DINKO instruments, Barcelona, Spain). Chloroform was used as a blank. Oxidation was reported as a function of the relative β-carotene content (% absorbance). Analyses were made in triplicate. 

## 3. Results and Discussion

### 3.1. Solution Properties

The successful development of encapsulation structures using electrospraying technology strongly depends on the solution properties and, hence, an initial characterization of solution viscosity and viscoelasticity was carried out. From a screening study, it was seen that HDPAF solutions led to very low apparent viscosity values at 5% (see Table 1) due to the low polymer (HDPAF) concentration, which resulted in unstable jetting and no capsules were formed from these solutions. In order to increase the viscosity of the solution, HDPAF concentration was increased. Viscosity values at 10% to 30% HDPAF concentration provided viscosity values previously reported as adequate for electrospraying (1 cP to 10 cP) [17].

Formulations with 40% of HDPAF produced a significant increase in viscosity. This could be attributed to the high amount of HDPAF added (40–50% *w*/*w*), but also to the high molecular weight of HDPAF, since they consist of a mixture of long polymers and fructooligosaccharides [6], which have been reported to contribute to increased viscosity [18]. In this case, the instability of the Taylor cone resulted in higher voltage values being needed to overcome the surface tension and achieve atomization. 

Similar values of surface tension in all formulations were obtained (Table 1). It was also observed that even though high HDPAF concentrations (30–50%) were used, the profile of surface tension values of the aqueous solutions decreased, but the range was still adequate. This behavior allows solutions and emulsions to be processed by electrospraying to obtain capsules. It has been previously reported that solutions with low surface tension favor the electrospraying process [20] and, thus, capsule formation [17], because the intensity of the electrical field must overcome the solution surface tension, expelling an electrified jet from the Taylor’s cone formed on the needle tip [21]. Therefore, during drying by electrospraying, the Taylor’s cone was held stable for formulations with low surface tension (~20 mN/m), which agrees with the work reported by Jaworek [22], who affirmed that solutions with surface tension above 50 mN/m cannot be electrosprayed, independently of the polymer used. This decrease in surface tension could be attributed to the ethanol addition to solubilize β-carotene in the formulations, because ethanol surface tension is lower than water surface tension [23]. The conductivity values increased when HDPAF increased from 5 to 20% in the solution, but conductivity decreased at concentrations of HDPAF above 30% (Table 1). However, conductivity values were always lower than values reported as adequate to be processed by electrospraying process (<2200.00 μS/cm) [18]. Finally, electrical conductivity should not exceed this value to avoid the destabilization of the electrospraying jet [23]. Emulsion and solution properties showed the same behavior with respect to the physical properties (viscosity, surface tension and conductivity) evaluated (Table 1). This can be attributed to the similar components in formulations. A technological advantage is that depending on the active compound polarity to be encapsulated, it can be selected between emulsions or solutions to incorporate as much compound as possible.

### 3.2. Capsule Morphology

**Figure 1:**
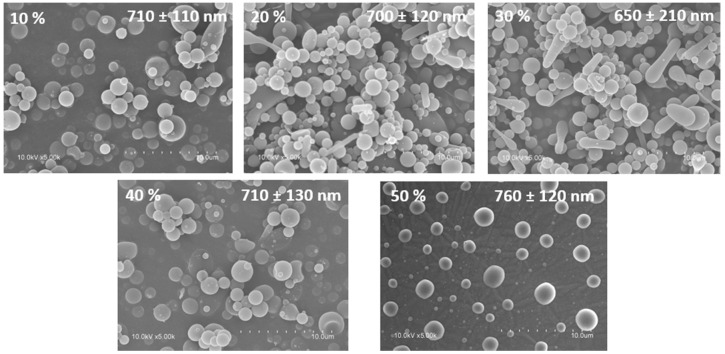
Micrographs obtained by scanning electron microscopy (SEM) of HDPAF nanocapsules at different HDPAF concentrations (% *w*/*w*: 10, 20, 30, 40 and 50) obtained by electrospraying.

SEM images demonstrated the ability of HDPAF to form capsules when HDPAF concentrations between 10% and 50% (*w*/*w*) were used as shown in Figure 1. Nanocapsules with spherical morphology and sizes between 650 nm and 760 nm, without cracks, dents or deformations and without being agglomerated (Figure 1) were obtained. The absence of pores or cracks on the capsule surface is important to ensure low oxygen permeability which could lead to the degradation of the encapsulated antioxidant compounds [24]. However, Ortiz-Basurto et al. [6] observed an irregular surface and several indentations on the microparticles of HDPAF encapsulating pitanga juice obtained by spray drying.

### 3.3. β-Carotene Encapsulation

The encapsulation of the β-carotene supposes a technical challenge due to its high instability to light, its hydrophobicity and its low solubility in common organic solvents [25,26,27]. The great interest of the food industry in this compound has motivated researchers to try the encapsulation of β-carotene by several methods. Tan and Nakajima [25] suggested the nanodispersion of β-carotene by the solvent evaporation method. Ribeiro et al. [26] proposed the encapsulation in Polylactide(PLA) and Poly lactic-*co*-glycolic acid (PLGA) by the solvent displacement method and Astete et al. [27] proposed the encapsulation in calcium alginate. However, some of these proposals presented the disadvantage of using organic solvents such as acetone [26], hexane [25] or chloroform [27]. Traces of these solvents would make the encapsulates unsuitable for food applications, and, therefore, it is of great interest to find a methodology to obtain nanocapsules based on the use of eco-friendly ingredients with a high encapsulation efficiency, which could reach the status of “generally recognized as safe (GRAS)” granted by the FDA.

Our first proposal was to use a solution to encapsulate the β-carotene. Nevertheless, the low solubility of β-carotene in conventional solvents prevented the obtaining of capsules with β-carotene concentrations over 0.1%. The second attempt was to use an emulsion, but for that option, the use of dichloromethane was required. The residual organic solvent concentration in the capsules was evaluated by headspace-solid-phase microextraction–gas chromatography (HS-SPME-GC) according to Camelo-Méndez et al., [28] with some modifications, and no traces of dichloromethane were detected in samples. Despite this good result, the concentration of the β-carotene in the particles was less than 1%. On the other hand, β-carotene encapsulated by the EC method allowed a higher concentration of β-carotene and consequently, the whole study was focused on this option.

### 3.4. FTIR Analysis of the Encapsulation Structures

**Figure 2:**
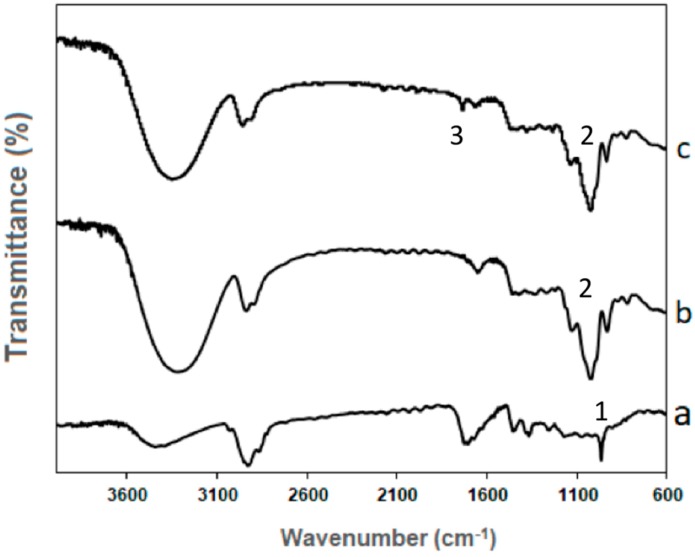
FTIR spectra. β-carotene (**a**), HDPAF nanocapsules (**b**) and HDPAF/β-carotene nanocapsules produced by the electrospraying coating (EC) process(**c**).

Interactions between β-carotene and HDPAF nanocapsules were evaluated by infrared spectroscopy (FTIR) according to Peinado et al. [29]. The FTIR spectra of β-carotene showed a broad peak at 3411 cm^−1^ that represents the presence of O–H stretching of the hydroxyl group, which is likely due to the interaction of β-carotene with oxygen in the air [30]. The peaks at 2929 cm^−1^ and 2869 cm^−1^ indicate the CH_2_ asymmetry and symmetry stretching, respectively (Figure 2a). The presence of carbonyl groups and the stretching symmetry of the C–H bond group was evidenced in peaks at 1717 cm^−1^ and 1366 cm^−1^, respectively. The sharp peak at 965 cm^−1^ marks the deformation mode of trans-conjugate alkenes as the specific areas of trans=CH (1 in Figure 2a) used for identification of β-carotene [30,31].

The FTIR spectra of HDPAF nanocapsules (Figure 2b) showed the most intensive broad band, with the maximum at 1050 cm^−1^ and two shoulders at 940 cm^−1^ and 1130 cm^−1^. The bands in the region 900–1153 cm^−1^ have been assigned to C–O and C–C stretching modes (2 in Figure 2b,c). These bands are characteristic of carbohydrates. Moreover, the two overlapped bands at 2930 cm^−1^ and 2870 cm^−1^ are characteristic of carbohydrates too [32]. The band from 2800 cm^−1^ to 3000 cm^−1^ is similar to the inulin spectra reported by Grube et al. [32] and Apolinário et al. [33]; this band is attributed to C–H stretching. The broad stretching peak around 3492 cm^−1^ indicated the presence of hydroxyl groups (–OH) of carbohydrates [33].

The comparison of nanocapsules of HDPAF and HDPAF/β-carotene obtained by the EC process (Figure 2b,c) proved HDPAF as the dominating component. The main differences in the nanocapsules of HDPAF and HDPAF/β-carotene spectra appeared in the 1700–1800 cm^−1^ region, which indicates the C=O interaction of fructose molecules with β-carotene, presenting as a stretching of the peak (3 in Figure 2c). The low intensity of β-carotene suggests that only a slight amount is located on the surface of the HDPAF nanocapsules [29]. Nanocapsules with a low surface intensity observed by FTIR (Figure 2c) suggest a centripetal distribution of β-carotene, where the highest concentration is in the core of the nanocapsule. Such confinement, likely due to the hydrophobicity of β-carotene, is desired, as it would create a barrier against oxygen and protection against thermal decomposition processes [29].

### 3.5. Thermal Stability of β-Carotene and HDPAF Nanocapsules

**Figure 3:**
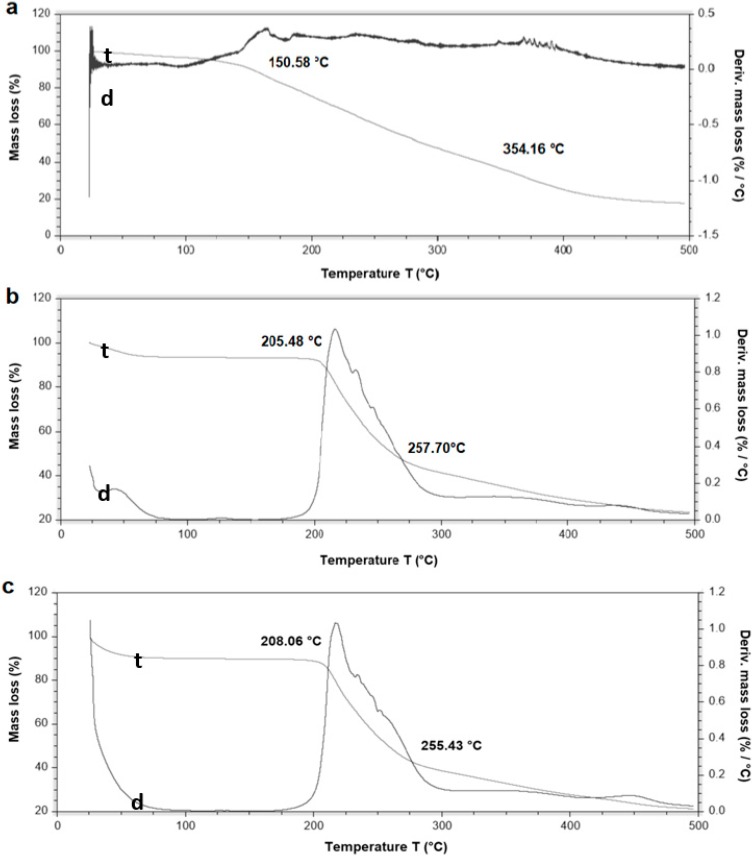
Thermogravimetric profile. β-carotene (**a**), HDPAF nanocapsules (**b**) and HDPAF/β-carotene nanocapsules obtained by EC (**c**). Curve t represents the thermogram and d the thermogram derivate.

The purpose of the thermogravimetric analysis was to evaluate the thermal resistance to degradation of β-carotene encapsulated in HDPAF nanocapsules. Thermograms shown, a termal decomposition of pure β-carotene between 150.58 °C and 354.16 °C (Figure 3a), similar decomposition temperature range (150–450 °C) was reported by Busolo and Lagaron (2015) [34]. HDPAF nanocapsules degraded between 205.48 °C and 257.70 °C (Figure 3b). These differences in stability can be associated to the structure of the molecules, since the HDPAF is a complex mixture of fructooligosaccharides [1,2] and may have functional properties linked to the degree of polymerization.

β-carotene encapsulated in HDPAF nanocapsules was decomposed between 208.60 °C and 255.43 °C (Figure 3c). This result supports the thermal protective effect of the HDPAF nanocapsule on β-carotene, similar to that reported by Peinado et al. [29] for the encapsulation of β-carotene in electrospun nanofibers of poly(ethylene oxide). The thermal stability of antioxidants as β-carotene depends on whether the molecules are totally encapsulated in the nanocapsules or on the surface [34]. Thermal stability of HDPAF/nanocapsules with and without β-carotene did not show a difference (Figure 3). Therefore, HDPAF exerts a protective role against the thermal degradation of β-carotene.

### 3.6. Ultraviolet (UV) Photostability of Encapsulated β-Carotene

**Figure 4:**
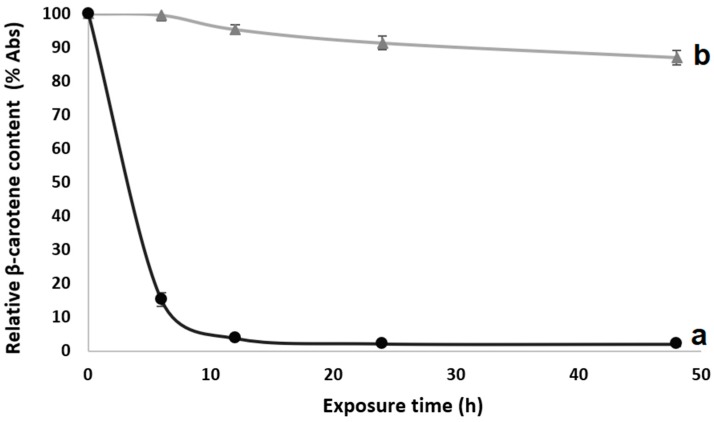
Relative decay in absorbance percentage (% Abs), as a function of exposure time to UV (a) β-carotene and (b) Nanoapsules with HDPAF and β-carotene by EC.

β-carotene is highly susceptible to photooxidation (oxidation or isomerization) due to the presence of conjugated double bonds in the molecule [19]. The exposure of β-carotene to UV light led to damage in the molecule, producing a decrease in the absorbance (measured at 466 nm) (Figure 4). Degradation of unprotected β-carotene has been also reported by Fernandez et al. [19] and de Freitas Zômpero et al. [21]. However, the β-carotene encapsulated in HDPAF showed a higher stability to UV light even after 48 h of exposure (Figure 4), attributed to the structure of the fructooligosaccharide mixtures.

Photoisomerization under UV light exposure is thought to be able to take place in free bioactive compounds, but not very readily in dried particles [19]. López-Rubio and Lagaron [35] produced hydrocolloid films (whey protein concentrate, zein, soy protein and gelatin) containing β-carotene which were able to maintain the β-carotene stability even after 50 h of UV light exposure. De Freitas Zômpero et al. [21] reported that a double encapsulation (nanoliposome + polymeric fiber) by electrospinning was useful to guarantee the β-carotene stability during 6 h of UV light exposure. Therefore, the utilization of the HDPAF as an encapsulating material could be a novel option to be used in nanocapsule manufacture to protect active compounds. In this case, the β-carotene loaded in HDPAF presented with similar behaviors when compared with other polymers/hydrocolloids used before [21,35]. However, this behavior was obtained with a low HDPAF concentration in the particle.

## 4. Conclusions

In this paper, high degree of polymerization Agave fructans (HDPAF) are presented as a novel encapsulating material. First, their ability to form capsules by electrospraying was tested. The best results, in terms of morphology and capsule size, were obtained when concentrations of 30% and 40% of fructans were used. β-carotene was encapsulated in HDPAF by direct electrospraying and by EC. However, the EC method presented advantages in comparison with emulsion or solution direct electrospraying, since it was possible to obtain particles with higher bioactive:polymer ratios. Moreover, the particles obtained by the EC method showed good photoprotection. Results shown in this work evidence that HDPAF have the capacity to improve the stability of β-carotene. Additionally, HDPAF are appropriate for human consumption, therefore they could be a really interesting encapsulation polymer for the food industry.

## Figures and Tables

**Table 1:** Physical properties (conductivity, surface tension, and viscosity) of solutions and emulsions at different high degree of polymerization Agave fructans (HDPAF) concentrations.

Concentration (% *w*/*w*)	Viscosity (cP)	Surface Tension (mN/m)	Conductivity (µS/cm)
**Solutions**			
5	1.61 ± 0.05 ^a^	24.35 ± 0.05 ^a^	69.39 ± 0.03 ^a^
10	2.37 ± 0.07 ^b^	24.37 ± 0.02 ^a^	82.14 ± 0.03 ^b^
20	3.42 ± 0.01 ^c^	24.85 ± 0.05 ^b^	101.20 ± 0.05 ^c^
30	6.82 ± 0.02 ^d^	23.65 ± 0.05 ^c^	93.30 ± 0.06 ^d^
40	46.05 ± 0.05 ^e^	23.51 ± 0.05 ^d^	76.73 ± 0.05 ^e^
50	162.22 ± 0.60 ^f^	23.46 ± 0.05 ^d^	52.86 ± 0.01 ^f^
**Emulsions**			
5	2.65 ± 0.03 ^a^	22.42 ± 0.04 ^a^	41.81 ± 0.04 ^a^
10	3.40 ± 0.03 ^b^	22.91 ± 0.02 ^b^	45.82 ± 0.04 ^b^
20	8.83 ± 0.08 ^c^	24.05 ± 0.03 ^c^	52.70 ± 0.02 ^c^
30	12.26 ± 0.09 ^d^	23.20 ± 0.02 _d_	50.89 ± 0.02 ^d^
40	45.70 ± 0.12 ^e^	22.73 ± 0.03 ^e^	39.11 ± 0.04 ^e^
50	93.54 ± 0.24 ^f^	21.13 ± 0.03 ^f^	30.26 ± 0.01 ^f^

^a–f^: Different superscripts within the same column indicate significant differences among the samples (*p* < 0.05).
